# Effects of Ionomycin on Egg Activation and Early Development in Starfish

**DOI:** 10.1371/journal.pone.0039231

**Published:** 2012-06-18

**Authors:** Filip Vasilev, Jong T. Chun, Giovanni Gragnaniello, Ezio Garante, Luigia Santella

**Affiliations:** Laboratory of Cellular and Developmental Biology, Stazione Zoologica Anton Dohrn, Villa Comunale, Napoli, Italy; Institute of Developmental Biology and Cancer Research, France

## Abstract

Ionomycin is a Ca^2+^-selective ionophore that is widely used to increase intracellular Ca^2+^ levels in cell biology laboratories. It is also occasionally used to activate eggs in the clinics practicing *in vitro* fertilization. However, neither the precise molecular action of ionomycin nor its secondary effects on the eggs' structure and function is well known. In this communication we have studied the effects of ionomycin on starfish oocytes and zygotes. By use of confocal microscopy, calcium imaging, as well as light and transmission electron microscopy, we have demonstrated that immature oocytes exposed to ionomycin instantly increase intracellular Ca^2+^ levels and undergo structural changes in the cortex. Surprisingly, when microinjected into the cells, ionomycin produced no Ca^2+^ increase. The ionomycin-induced Ca^2+^ rise was followed by fast alteration of the actin cytoskeleton displaying conspicuous depolymerization at the oocyte surface and in microvilli with concomitant polymerization in the cytoplasm. In addition, cortical granules were disrupted or fused with white vesicles few minutes after the addition of ionomycin. These structural changes prevented cortical maturation of the eggs despite the normal progression of nuclear envelope breakdown. At fertilization, the ionomycin-pretreated eggs displayed reduced Ca^2+^ response, no elevation of the fertilization envelope, and the lack of orderly centripetal translocation of actin fibers. These alterations led to difficulties in cell cleavage in the monospermic zygotes and eventually to a higher rate of abnormal development. In conclusion, ionomycin has various deleterious impacts on egg activation and the subsequent embryonic development in starfish. Although direct comparison is difficult to make between our findings and the use of the ionophore in the *in vitro* fertilization clinics, our results call for more defining investigations on the issue of a potential risk in artificial egg activation.

## Introduction

Fertilized eggs undergo a series of rapid changes such as rearrangement of the cytoskeleton, alteration of the electrical property of the plasma membrane, coordinated exocytosis of cortical granules, and initiation of DNA replication and protein synthesis [1], [2]. In virtually all animal species, these metabolic and cytological changes, collectively termed ‘egg activation,’ are accompanied by a substantial increase of the intracellular Ca^2+^ that propagates through the cytoplasm as a single or oscillating wave [3]–[5]. The demonstration that nearly all aspects of egg activation can be recapitulated by artificially increasing the intracellular Ca^2+^ levels has led to the prevailing view that Ca^2+^ serves as a master key to initiate all these cytological changes in the fertilized eggs [6], [7], although Ca^2+^-independent pathways might also exist and contribute to egg activation [8].

In physiological conditions, intracellular Ca^2+^ level can be increased either by influx from the extracellular space or by release from the intracellular stores. It has been demonstrated that Ca^2+^-linked second messengers such as InsP_3_ (inositol 1,4,5-trisphosphate), cADPr (cyclic ADP ribose) and NAADP (nicotinic acid adenine dinucleotide phosphate) are the mediators of the intracellular Ca^2+^ release in response to various stimuli [9]–[11], and these second messengers may have distinct roles in creating and propagating Ca^2+^ waves inside fertilized eggs [12]. However, intracellular Ca^2+^ levels can also be conveniently elevated by use of ionophores. Ionomycin is a widely used Ca^2+^-selective ionophore that has been isolated from the bacterium *Streptomyces conglobatus* [13]. This mobile ion carrier binds Ca^2+^ in one-to-one stoichiometry and promotes mostly electrically neutral exchange of Ca^2+^ for 2H^+^ or other divalent cations such as Mg^2+^, and thereby transports Ca^2+^ ions across the vesicle membranes or through the water-lipid interface [14], [15]. As a Ca^2+^-selective ionophore, ionomycin is known to be more specific and potent than A23187 [16], but the exact mechanism by which it raises the Ca^2+^ levels inside an integral living cell is not fully understood and still remains controversial [17]. Extending the findings from the vesicle membranes, ionomycin may directly target the plasma membrane and induce Ca^2+^ influx, or act on the intracellular organelles to release Ca^2+^. Alternatively, it may take an indirect route either to potentiate the existing Ca^2+^-mobilizing mechanisms or to activate other enzymes such as phospholipase C (PLC) that in turn produces the Ca^2+^-mobilizing second messenger InsP_3_. The alternative actions of ionomycin might depend on its concentration, as ionomycin has predominantly ionophoretic effects at >1 µM [17], [18].

As an experimental model, starfish provide a synchronous population of oocytes that are arrested at the prophase of the first meiotic division. These immature oocytes characteristically containing a large nucleus termed ‘germinal vesicle (GV)’ can be induced to reenter the cell cycle (meiotic maturation) by exposure to the maturation hormone, 1-methyladenine (1-MA). At the onset of meiotic maturation, starfish oocytes readily (<2 min after 1-MA addition) releases Ca^2+^ from internal stores [19]–[21] and slowly relocate cortical granules toward the plasma membrane by an actin-dependent mechanism, as was also demonstrated in sea urchin [22], [23]. However, the physiological significance of this Ca^2+^ mobilization has not been fully established because artificial elevation of intracellular Ca^2+^ levels with A23187 did not induce maturation by itself [24]. Nonetheless, inhibition of the natural occurrence of the Ca^2+^ signals with calcium chelators blocked GV breakdown and the meiotic progress in starfish oocytes [20]. A possible explanation reconciling these two seemingly conflictive results would be that, unlike egg activation, triggering the adequate cytological changes at meiotic maturation might require highly delicate intracellular Ca^2+^ signaling that cannot be mimicked by the Ca^2+^ ionophore, as was exemplified in the Ca^2+^-dependent cytoskeletal changes in neuronal growth cones [25]–[27]. In addition, an imprecise Ca^2+^ increase by ionophores might have caused other structural and functional changes in cells.

In this communication, we have studied the effects of ionomycin on egg activation and subsequent development in starfish. Since ionomycin by itself activates mature eggs, we have used the GV stage oocytes briefly exposed to the ionophore, and followed the consequences in meiotic maturation, fertilization, and early development. We observed that artificial elevation of intracellular Ca^2+^ levels with ionomycin immediately led to drastic alteration of the actin cytoskeleton in the immature oocytes, which exhibited depolymerization of the actin meshwork in the subplasmalemmal regions along with highly enhanced polymerization and bundling of the actin filaments in the inner cytoplasm. These cytoskeletal changes were accompanied by retraction of the microvilli at the oocyte surface and the loss of cortical granules that were either engulfed by other vesicles or exocytosed to the perivitelline space, which mimicked the changes at egg activation. Ionomycin neither provoked nor inhibited breakdown of the nuclear envelope, which is the hallmark of the meiotic maturation. However, the oocytes' history of being briefly exposed to ionomycin prevented completion of the other processes of meiotic maturation: the structural changes in the ectoplasm of the oocytes [28], [29]. As a result, the mature eggs that had been briefly exposed to ionomycin at the GV stage responded to fertilizing sperm and InsP_3_ with no elevation of the fertilization envelope. The monospermic zygotes derived from these eggs tended to have problems in cell cleavage and showed a higher rate of abnormal development. The detrimental effect of ionomycin on the early stage of development was also confirmed in the monospermic zygotes briefly exposed to ionomycin.

## Results

### Ionomycin increases intracellular Ca2+ levels in the immature oocytes of starfish by mobilizing Ca2+ from intracellular stores and by Ca2+ influx

**Figure 1:**
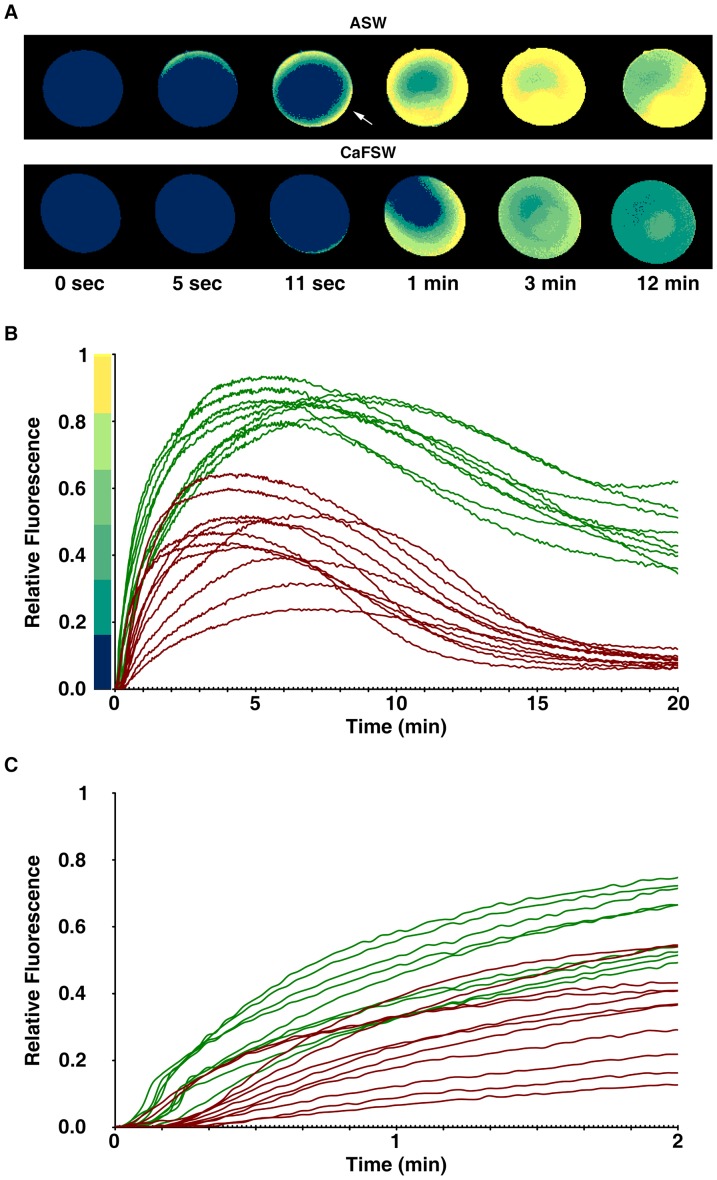
Changes of intracellular Ca^2+^ levels in the starfish oocytes exposed to ionomycin. *A. aranciacus* oocytes at the GV stage were microinjected with Calcium Green/Rhodamine Red and subsequently exposed to 5 µM ionomycin in artificial seawater in the presence (ASW) or absence (CaFSW) of 10 mM Ca^2+^. Ca^2+^ images were then captured with epifluorescence microscopy as described in Materials and Methods. (**A**) The pseudocolored images of Ca^2+^ changes within the representative oocytes at several key time points. Indicated by an arrow is the cortical flash. (**B**) The trajectories of the Ca^2+^ responses quantified at the entire cytoplasmic field. The Ca^2+^ responses in ASW and CaFSW are represented in green and brown curves, respectively. To compare the kinetics of the Ca^2+^ rises in ASW and CaFSW, the moment of the first detectable Ca^2+^ signal was set to t = 0 in panels A and B. (**C**) The initial response of the oocytes to ionomycin in ASW (green curves) and CaFSW (brown curves). To better illustrate the difference in the time lag before the first detectable Ca^2+^ rise, the moment of the ionomycin addition was set to t = 0 in this panel.

Live oocytes of *Astropecten aranciacus* were microinjected with Calcium Green and Rhodamine Red and examined with the CCD camera to monitor the changes of cytosolic Ca^2+^ levels in response to 5 µM ionomycin. In the artificial seawater containing 10 mM Ca^2+^ (ASW), oocytes promptly started to increase the intracellular Ca^2+^ level after the addition of ionomycin. The Ca^2+^ signal surpassed half the maximal level within 90 sec and arrived at the plateau by 5 min (Fig. 1B). The Ca^2+^ signals were initially prominent in the cortical area before spreading to the center of oocytes (Fig. 1A), and eight out of nine oocytes displayed a sharp rise and fall of Ca^2+^ signals in the entire cortical regions subjacent to the plasma membrane (cortical flashes) at the initial stage of the Ca^2+^ rise (10.5±2.9 sec) (Fig. 1A, arrow). In the Ca^2+^-free seawater (CaFSW), however, the Ca^2+^ response came out with a significantly longer latent period after the addition of ionomycin (16.2±5.3 sec, n = 11) in comparison with the oocytes in ASW (6.7±2.9 sec; n = 9, *p*<0.0001), as shown in Fig. 1C. In line with the idea that the short-lived (<2 sec) cortical flash represents a Ca^2+^ influx from outside [30], [31], the same concentration of ionomycin (5 µM) produced no cortical flash (0 out of 11) in the CaFSW (Fig. 1A). In the absence of extracellular Ca^2+^, the ionomycin-exposed oocytes released Ca^2+^ from the internal stores, but the amplitude of the Ca^2+^ peak was significantly lower (0.46±0.12 RFU, n = 11) than that in the Ca^2+^-containing seawater (0.86±0.04 RFU, n = 9; *p*<0.0001). In addition, the Ca^2+^ level inside the oocytes exposed to 5 µM ionomycin in CaFSW fell to the basal level within 15 min (Fig. 1B), whereas the oocytes in the ASW containing 5 µM ionomycin displayed the declining Ca^2+^ signals still being maintained at much elevated levels (Fig. 1B). Hence, while both external and internal Ca^2+^ stores contribute to the Ca^2+^ rise in response to ionomycin, full-fledged and prolonged elevation of cytosolic Ca^2+^ requires external Ca^2+^.

### Ionomycin induces retraction of microvilli and fusion of cortical vesicles in the immature oocytes and eggs of starfish

**Figure 2:**
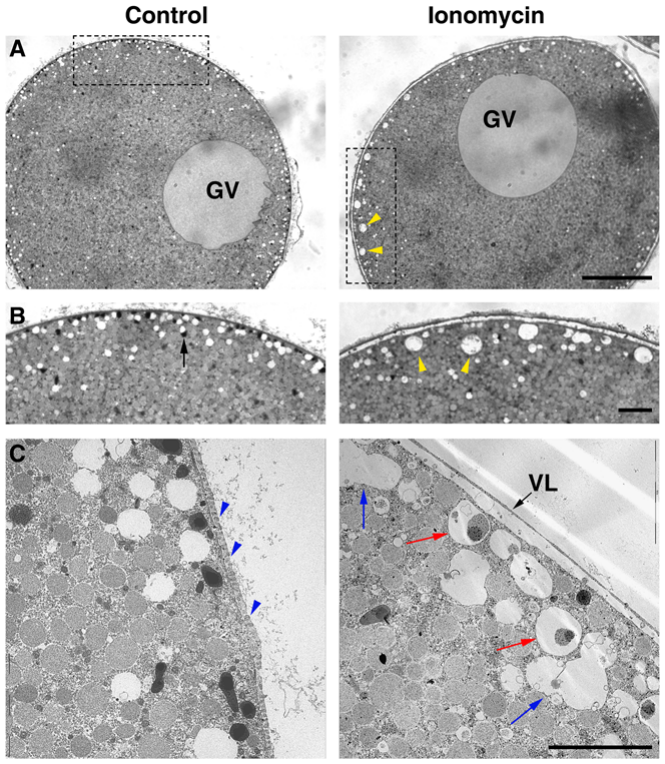
Morphological changes in the cortex of the starfish oocytes exposed to ionomycin. *A. aranciacus* oocytes at the GV stage were fixed in glutaraldehyde after 5 min incubation with 5 µM ionomycin in natural seawater. (**A**) Bright field view in the light microscope. GV = germinal vesicle. Scale bar = 50 µm. (**B**) The magnified views of the dot-lined rectangular areas in panel A. The same large vesicles in panel A were marked with yellow arrowheads. Note that cortical granules that appear as dark vesicles sized about 1 µm (arrow) had largely disappeared in the oocytes briefly exposed to ionomycin. Scale bar = 10 µm. (**C**) TEM image of the same batch of oocytes incubated in the absence (left) or present of 5 µM ionomycin for 5 min. Blue arrowheads indicate microvilli in cross-section. Red arrows indicate the white vesicles engulfing electron-dense cortical granules. Blue arrows, white vesicles at fusion; Scale bar = 10 µm.

We noted that the starfish oocytes at the GV stage often responded to ionomycin with slight elevation of the vitelline layer (Fig. 2), as was previously reported in the context of ectoplasmic maturation in the oocytes exposed to sub-threshold dose of the maturation hormone [28], [29]. Furthermore, we noted that the ‘white vesicles’ that are normally present in the ectoplasm of the oocytes under the light microscope are much reduced in number but increased in size in the oocytes exposed to 1–5 µM ionomycin (Fig. 2A and B, yellow arrowheads). The white vesicles in the control oocytes are rather homogenous in size, averaging 1.2 µm in diameter. On the other hand, the size of the ‘large white vesicles’ in the ionomycin-treated oocytes reached 4–8 µm, when measuring the longer axis. In support of the idea that the occurrence of the bigger white vesicles is due to intervesicular fusion, the TEM (transmission electron microscopy) image clearly showed two intermediately large vesicles (>4 µm) forming a ‘peanut shell-shaped’ twin structure in the ionomycin-pretreated oocytes (Fig. 2C, blue arrows). Interestingly, subjacent to the partially elevated vitelline layer, the white vesicles appeared to have engulfed the electron-dense materials that presumably derived from the cortical granules (Fig. 2C, red arrows). Furthermore, a myriad of finger-like protruding structures that are filled with actin filaments on the surface of the control oocytes (microvilli: Fig. 2C, blue arrowheads) were largely absent in the ionomycin-treated oocytes. As a result, the partially elevated vitelline layer left behind a smooth plasma membrane without revealing any sign of microvilli in the perivitelline space. Amid the disruption of the cortical granules and the fusion of white vesicles, other vesicles such as yolk platelets, which appear as most numerous and intermediately dark granules in the TEM (Fig. 2C), did not undergo much structural change. Hence, it appears that ionomycin selectively targets white vesicles and cortical granules but spares other vesicles and organelles. At the same time, microvilli were mostly retracted by the same ionomycin treatment.

### Ionomycin induces drastic rearrangement of the actin cytoskeleton in the immature oocytes and eggs of starfish

**Figure 3:**
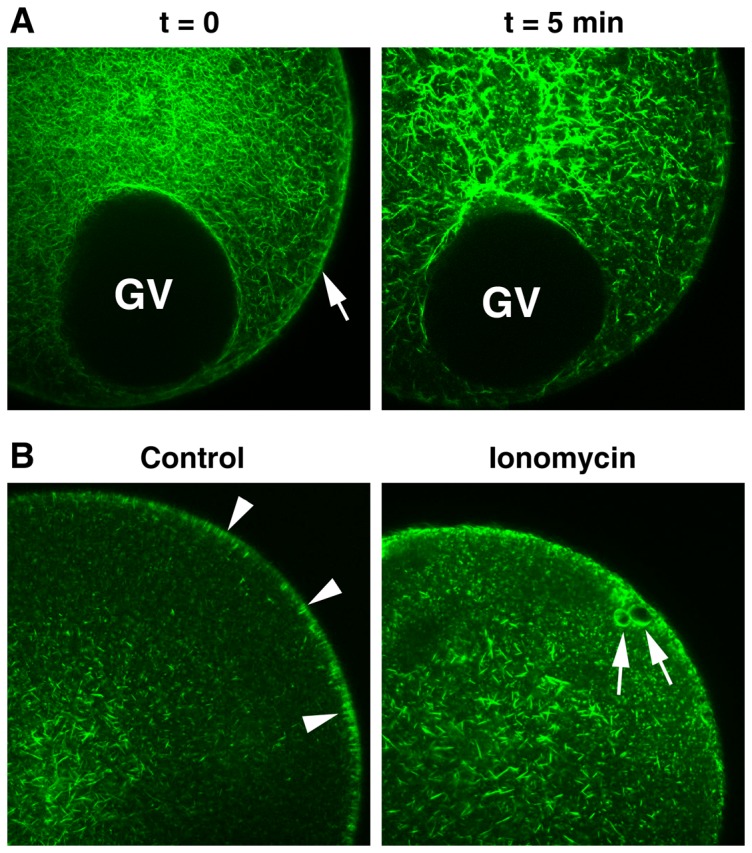
Ionomycin induces rapid rearrangement of the actin cytoskeleton. (**A**) A living oocyte (*A. aranciacus*) microinjected with Alexa Fluor 488-conjugated phalloidin was exposed to 5 µM ionomycin and monitored under the confocal microscope. Note the continuous layer of the subplasmalemmal actin network delineating the plasma membrane before the ionomycin treatment (arrow, t = 0) had mostly disappeared within 5 min in the same oocytes. In contrast, the actin filaments in the inner cytoplasm formed bundles and became much thicker and longer. (**B**) After the brief exposure (5 min) to 5 µM ionomycin, the oocytes were switched to normal seawater without ionomycin and induced to undergo meiotic maturation in the presence of 10 µM 1-MA for 1 hour. The orderly arranged actin filaments seen in the control eggs (arrowheads) are mostly lost in the eggs briefly exposed to ionomycin at the GV stage. Instead, a thick layer of actin fibers surrounded the big fused white vesicles (arrows).

As demolition of microvilli on the oocyte surface implied rapid depolymerization of microfilament bundles within, we examined the structural changes of the actin cytoskeleton inside a living oocyte after the ionomycin treatment. Under the confocal microscope, the GV stage oocytes microinjected with the Alexa Fluor 488-conjugated phalloidin revealed dramatic alteration of the actin-cytoskeleton after 5 minutes' exposure to 5 µM ionomycin (Fig. 3A), whereas F-actin structures of the untreated oocytes remained virtually unchanged within the same timeframe (not shown). Thus, during the period of ionomycin-induced Ca^2+^ rise, the actin cytoskeleton underwent highly accelerated rearrangement. Specifically, in the inner cytoplasm, the actin cytoskeleton became extensively polymerized, forming much longer and thicker microfilament bundles and patches. In the subplasmalemmal region, however, the typical actin filaments that are intimately associated with the plasma membrane (Fig. 3A, white arrow at t = 0) conspicuously disappeared by 5 min, indicating active depolymerization of the actin filaments in this subcellular domain. This striking structural rearrangement of the actin filaments in the subplasmalemmal area and the inner cytoplasm were maintained for more than 20 min as long as ionomycin was kept in the media (data not shown). Hence, ionomycin had dual effects on the actin cytoskeleton remodeling: fast and extensive depolymerization in the subplasmalemmal region and the simultaneous polymerization in the inner cytoplasm. When the GV stage oocytes briefly (5 min) exposed to ionomycin were washed and switched to normal seawater for >1 h in the presence of 1-MA (10 µM), the actin cytoskeletal structure was, to some extent, restored to the state of the control mature eggs (Fig. 3B). However, in these eggs, the organization of the F-actin bundles that are perpendicular to the plasma membrane in the control eggs (Fig. 3B arrowheads) were largely absent despite the restoration of the dense actin meshwork in the region. Interestingly, the borders of the large vesicles formed by ionomycin treatment were vested with a thick layer of actin fibers (Fig. 3B, arrows). Despite these structural changes in the ectoplasm, the eggs that had been pretreated with ionomycin at the GV stage underwent apparently normal meiotic maturation in the nucleus, displaying GV breakdown in the same time schedule (1 h) after 1-MA addition.

### Brief exposure of the GV stage oocytes to ionomycin induces disruption and exocytosis of cortical granules

**Figure 4:**
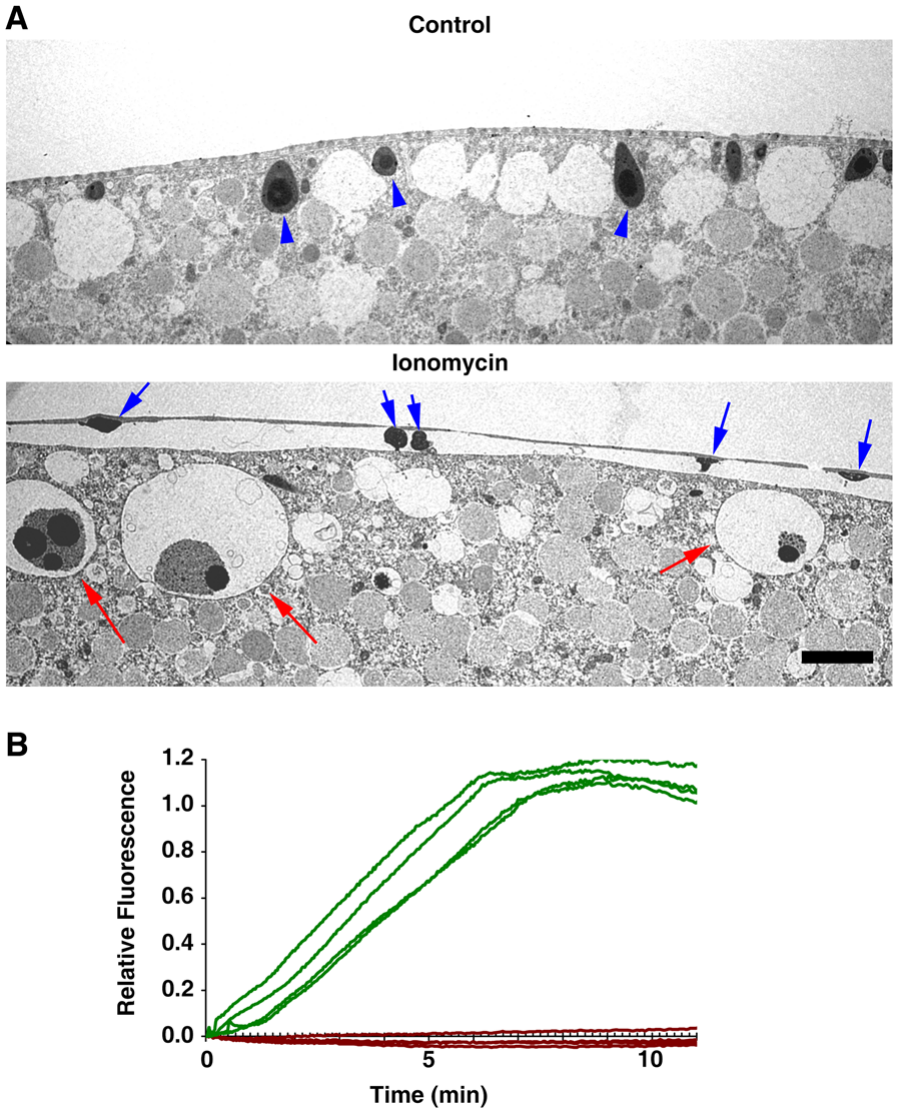
Disruption of cortical granules and microvilli by the brief exposure to ionomycin leads to depletion of the ionomycin-sensitive Ca^2+^ stores. *A. aranciacus* oocytes at the GV stage were exposed to 5 µM ionomycin for 3 min before switched to the media containing 1-MA. (**A**) After 1 h incubation, the mature eggs were fixed with glutaraldehyde and analyzed by TEM. Blue arrows indicate the remnant of the cortical granules that were extruded in the perivitelline space. Red arrows indicate fragments of cortical granules being engulfed by white vesicles. Scale bar = 10 µm. (**B**) The same batch of oocytes were exposed to 5 µM ionomycin for 3 min and switched to the fresh media containing 1-MA. After GV breakdown, the mature eggs were microinjected with Calcium Green/Rhodamine Red and subsequently re-exposed to 5 µM ionomycin (t = 0) to monitor the Ca^2+^ response. The trajectory of intracellular Ca^2+^ levels in the eggs with or without (control) ionomycin pretreatment were depicted in brown and green curves, respectively.

**Figure 5:**
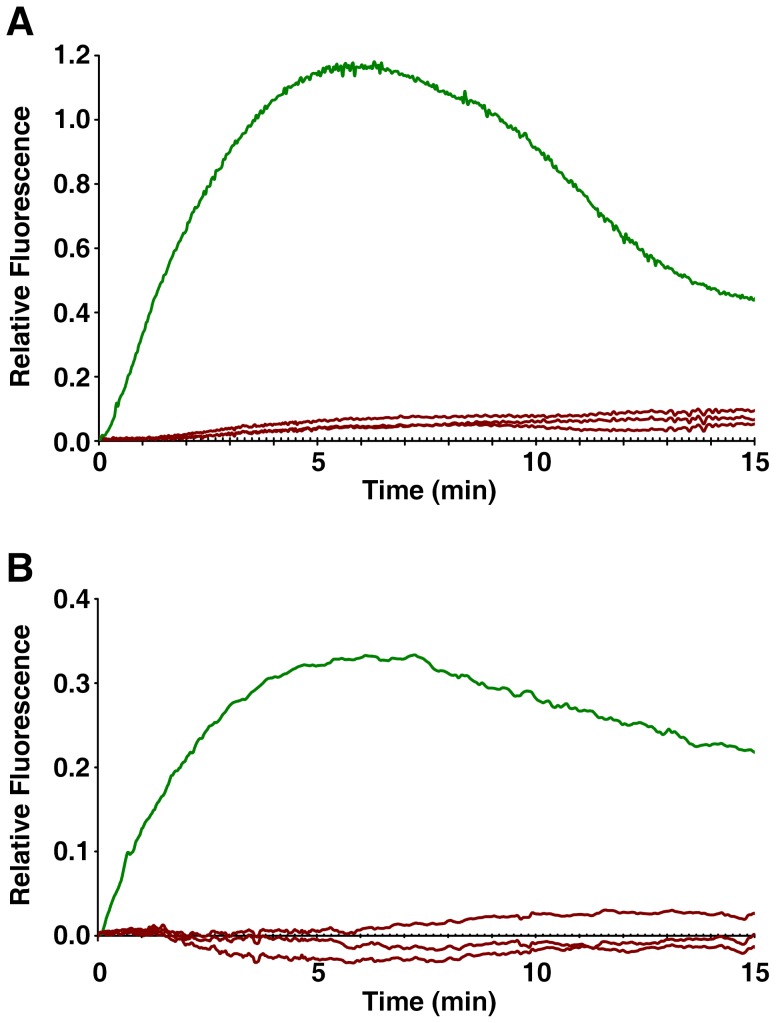
The mature eggs pretreated with ionomycin at the GV stage respond to the second dose of ionomycin or A23187 with no intracellular Ca^2+^ increase. *P. miniata* oocytes at the GV stage were briefly exposed to 5 µM ionomycin and switched to the normal seawater containing 10 µM 1-MA for 1 h and subsequently challenged with the second dose of 5 µM ionomycin (**A**) or 40 µM A23187 (**B**). In both cases, the green curves depict the Ca^2+^ response in the control eggs, and the brown ones the response of the eggs that had been briefly exposed to 5 µM ionomycin at the GV stage.

In parallel with the general propensity for actin depolymerization in the subplasmalemmal region (Fig. 3), the oocytes briefly (3–5 min) exposed to 5 µM ionomycin before the addition of maturation hormone 1-MA displayed signs of cortical granules exocytosis and disruption. Whereas the control eggs displayed typical arrangement of cortical granules closely attached to the plasma membrane at the end of maturation (Fig. 4A, arrowheads), the eggs that had been briefly exposed to ionomycin before undergoing maturation process showed much reduced number of cortical granules. It appears that those cortical granules that were already at the vicinity of the plasma membrane underwent exocytosis (Fig. 4A, blue arrows), while the other ones that had not been yet translocated toward the plasma membrane were engulfed by white vesicles (Fig. 4A, red arrows). This is in sharp contrast with the control eggs in which cortical granules remain intact despite the presence of vicinal white vesicles. It is also noteworthy that the ultrastructure of the cortices of the oocytes exposed to 5 µM ionomycin were not altered by further exposure to 1-MA (compare the TEM images of the ionomycin-exposed oocyte and egg in Fig. 2C and 4A). Hence, the brief exposure to ionomycin inflicted largely irreversible structural changes in the subplasmalemmal region. Indeed, despite the fact that the depolymerized actin reassembled the actin meshwork, albeit irregular, subjacent to the plasma membrane (Fig. 3B), the microvilli were still totally absent in these eggs (Fig. 4A). Interestingly, when exposed again to ionomycin after maturation, these eggs no longer produced intracellular Ca^2+^ release (Fig. 4B, brown curves) unlike the control eggs that had not been pretreated with 5 µM ionomycin at the GV stage (Fig. 4B, green curves). The same result was also obtained with the oocytes of a different starfish species, *Patiria miniata* (also known as *Asterina miniata*). As shown in Fig. 5A, a brief exposure to 5 µM ionomycin at the GV stage prevented the mature eggs from responding to ionomycin with intracellular Ca^2+^ increase. The failure of the second dose of ionomycin to evoke a Ca^2+^ response is not due to the potential acidification of the eggs and the reported inefficacy of ionomycin at low pH; unlike ionomycin that has no Ca^2+^-complexing activity below pH 7.0, A23187 maintains its Ca^2+^ ionophore activity at pH 5 to 10 [14]. With 40 µM A23187, which induced intracellular Ca^2+^ increase in control eggs, the ionomycin-pretreated eggs at the GV stage did not mobilize Ca^2+^ (Fig. 5B). Taken together, these results suggest that the structural changes inflicted by the brief exposure to ionomycin, i.e. elimination of microfilaments-filled microvilli and disruption of the cortical granules and the fusion of white vesicles, are linked to the depletion of the ionomycin-sensitive Ca^2+^ stores.

### Ionomycin-sensitive Ca^2+^ pool in the cortex of starfish eggs

**Figure 6:**
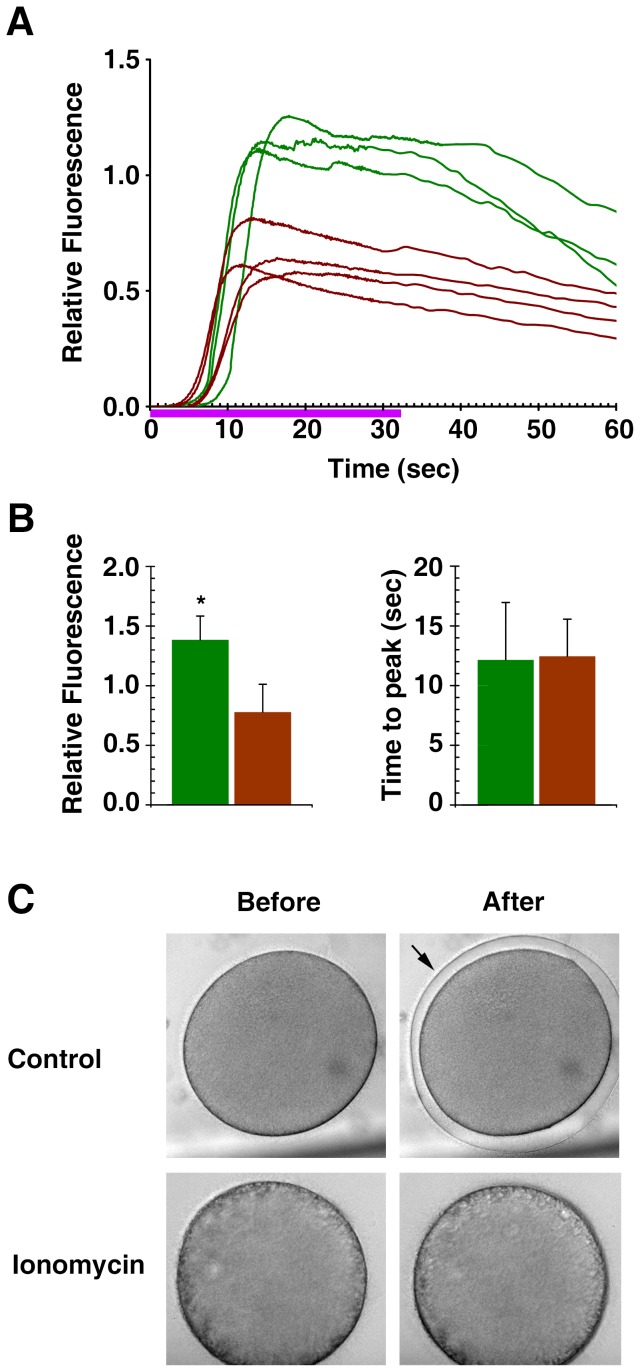
Ionomycin-exposed eggs with cortical granule disruption still respond to InsP_3_ with an intracellular Ca^2+^ release, but to a reduced extent. *A. aranciacus* oocytes were exposed to 5 µM ionomycin for 5 min at the GV stage and microinjected with caged InsP_3_ and Calcium Green. The oocytes were matured in the fresh seawater containing 10 µM 1-MA and then irradiated with UV to photoactivate the Ca^2+^-mobilizing second messenger. (**A**) Results of one of the three independent experiments showing the trajectories of the quantified Ca^2+^ responses at the entire cytoplasmic field. Ca^2+^ responses in the control eggs and the eggs briefly exposed to 5 µM ionomycin at the GV stage were shown in green and brown curves, respectively. Violet line indicates the duration of UV irradiation. (**B**) Summary of the data pooled from three independent batches of experiments comprising 3 or 4 microinjected eggs with (brown bars, n = 10) or without (control, green bars; n = 9) ionomycin pretreatment at the GV stage. The average amplitude (mean ± standard deviation, left histogram) and the time interval between the onset and the peak of the Ca^2+^ signals (right histogram) were depicted separately. Asterisk indicates a significant difference between the control and the ionomycin-pretreated eggs (*p*<0.0001). (**C**) Despite the substantial amount of Ca^2+^ released, the ionomycin-pretreated eggs did not undergo elevation of the vitelline layer in all cases.

**Figure 7:**
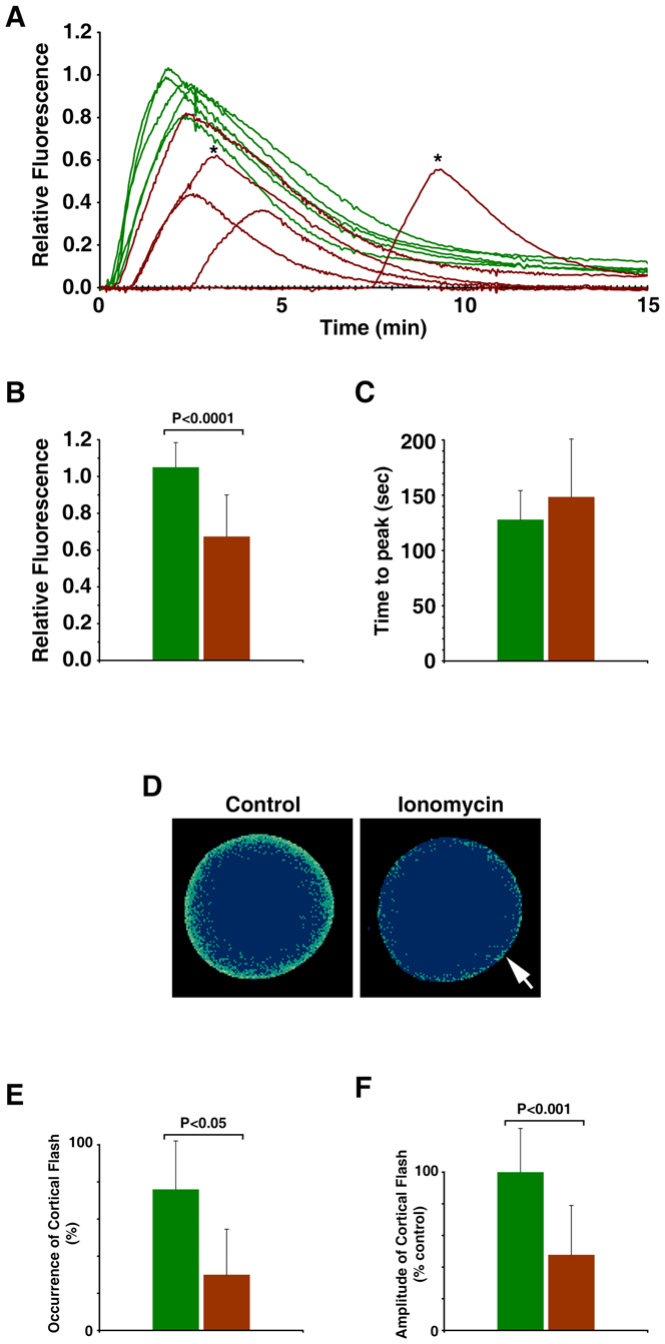
Fertilization of the ionomycin-pretreated eggs with altered cortical structure. *A. aranciacus* oocytes were briefly exposed to ionomycin (5 µM for 5 min) at the GV stage and subsequently incubated in fresh seawater containing 10 µM 1-MA. The mature eggs were then inseminated. (**A**) Results of one of the five independent experiments. The trajectories of the quantified Ca^2+^ responses at the entire cytoplasmic field. Ca^2+^ responses in the control eggs and the eggs briefly exposed to 5 µM ionomycin at the GV stage were shown in green and brown curves, respectively. To illustrate the difference in the latent period before the Ca^2+^ response, the moment of the fertilizing sperm addition was set to t = 0. Asterisks indicate the Ca^2+^ peaks of the eggs that required a second addition of sperm (5 min after the first insemination). (**B–F**) Summary of the data pooled from five independent batches of experiments comprising 4 to 8 eggs with (brown bars, n = 20) or without (control, green bars; n = 23) ionomycin pretreatment at the GV stage. The average amplitude (mean ± standard deviation) of the Ca^2+^ peaks and the time interval between the onset and the peak of the signals were plotted in panels **B** and **C**, respectively. (**D**) Pseudocolor images of the representative cortical flashes in the control and the ionomycin-pretreated eggs (arrow) from the same batch of experiment. Ca^2+^ images were captured with epifluorescence microscopy as described in the Materials and Methods. (**E**) Frequency of the detectable cortical flashes in the same five independent experiments. (**F**) Comparison of the amplitude of the cortical flashes. Data were normalized in reference to the average value of the control eggs in each batch of experiment.

When ionomycin-pretreated oocytes were switched back to normal seawater and induced to mature for 1 h in the presence of 1-MA, the cytosolic Ca^2+^ returned to the basal level of the control mature eggs that had not been exposed to ionomycin (data not shown). Thus, the mobilized Ca^2+^ had mostly returned or was at least en route to its original stores. Although not responding to the second exposure to ionomycin nor A23187 with Ca^2+^ release (Fig. 4 and 5), these ionomycin-pretreated eggs still responded to InsP_3_ with a substantial release of Ca^2+^ (Fig. 6). The amplitude of the Ca^2+^ peak in these eggs (0.78±0.23 RFU, n = 10) was consistently lower (by 43%) than that of the control mature eggs (1.38±0.20 RFU, n = 9; *p*<0.0001). Thus, the alteration of the egg cortex, e.g. retraction of microvilli and disruption of cortical granules (Fig. 4A), was associated with the reduction of the amplitude of the intracellular Ca^2+^ release in response to InsP_3_. However, the kinetics of the Ca^2+^ rise was not changed by the ionomycin treatment, as the time required to reach the Ca^2+^ peak remained virtually the same in the control (12.2±4.8 sec, n = 9) and the ionomycin-pretreated eggs (12.5±3.1 sec, n = 10; *p* = 0.8681) (Fig. 6B, right panel). It is noteworthy that the eggs that had been briefly exposed to ionomycin at the GV stage did not elevate the vitelline layer despite the substantial increase of intracellular Ca^2+^ in all cases (Fig. 6C). In addition, for these eggs to produce the characteristic fertilization Ca^2+^ wave, repeated insemination was required in the majority of cases (Fig. 7A). Even when the ionomycin-pretreated eggs responded at once to a single sperm, the onset of the Ca^2+^ wave was much delayed (68.7±64.3 sec after sperm addition, n = 3) in comparison with the control eggs in the same batch of experiment (19.2±9.2 sec, n = 10; *p*<0.05). Similar to the results obtained with uncaged InsP_3_, the peak amplitude of the sperm-induced Ca^2+^ transient in the eggs that had been exposed to ionomycin at the GV stage was about 35.9% lower (0.67±0.23 RFU, n = 20) than that of the control eggs (1.05±0.13 RFU n = 23; *p*<0.0001) (Fig. 7A and B). Again, however, the kinetics of the Ca^2+^ rise was not significantly altered, as the time required for reaching the peak in the ionomycin-pretreated eggs (148.4±52.4, n = 20) was not significantly different from that in the control (128.0±26.1 sec, n = 23; *p* = 0.1061), implying that the intracellular mechanism that sustain the propagation of the Ca^2+^ wave remained intact in these eggs (Fig. 7B). Another conspicuous feature of the fertilization in the ionomycin-pretreated eggs is that the cortical flash that normally occurs at the very beginning of fertilization in this species [10] is often missing or substantially reduced in its amplitude. In five independent experiments, the incidence of the cortical flash in the ionomycin-pretreated eggs (30±24%, n = 5) was greatly reduced from the average values in the corresponding control eggs (76±2%, n = 5; *p*<0.05) (Fig. 7E and F). The amplitude of the cortical flash displayed by the ionomycin-pretreated eggs was also significantly reduced, corresponding to merely 47.8±31.2% (n = 7) of the values in the control (100±27.6%, n = 17; *p* = 0.0005) (Fig. 7D and E). Hence, the results on the cortical flash again indicated that the subcellular events highly restricted to the egg surface were substantially influenced by the pretreatment of the oocytes with ionomycin.

**Figure 8:**
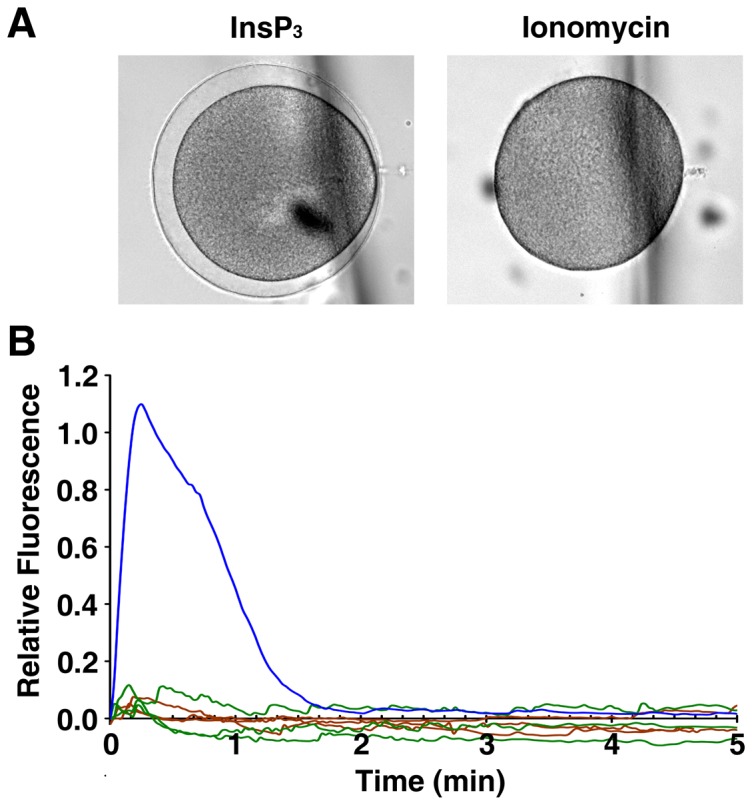
Microinjected ionomycin does not induce Ca^2+^ increase inside the starfish eggs. *P. miniata* oocytes were microinjected with Calcium Green and induced to mature in 10 µM 1-MA for 1 h. Under the CCD camera, the mature eggs were microinjected with InsP_3_ (without caging, 5 µM in pipette tip), ionomycin (50 µM), or the injection buffer only. Results of one of the three independent experiments are shown. (**A**) Transmission views of the eggs 10 min after microinjection. (**B**) Quantified Ca^2+^ signals for InsP_3_ (blue curve), injection buffer (green), and ionomycin (brown).

Ionomycin-pretreated eggs responding to InsP_3_ and fertilizing sperm (Fig. 6 and 7) but not to the second dose of ionomycin (Fig. 4 and 5) raises a possibility that the cation-complexing agent does not reach deep down to the inner cytoplasm of big cells like starfish eggs in bath incubation. To test if the lack of Ca^2+^ response is due to this, we have microinjected the eggs with ionomycin. To our surprise, not only the ionomycin-pretreated eggs but also the control eggs responded to the microinjected ionomycin with no significant Ca^2+^ increase with 50 µM of ionomycin in the injection pipette. This is not due to a technical problem related to the simultaneous microinjection and Ca^2+^ detection because the parallel experiments with InsP_3_ microinjection (5 µM in the injection pipette) using the same method produced high level of Ca^2+^ response as was expected (Fig. 8). Microinjected ionomycin did not induce any increase of Ca^2+^ even with 10 times higher dose (500 µM in the injection pipette), and the same negative result was observed with the immature oocytes (not shown).

### Fertilization and early development of the starfish eggs exposed to ionomycin at the GV stage

**Figure 9:**
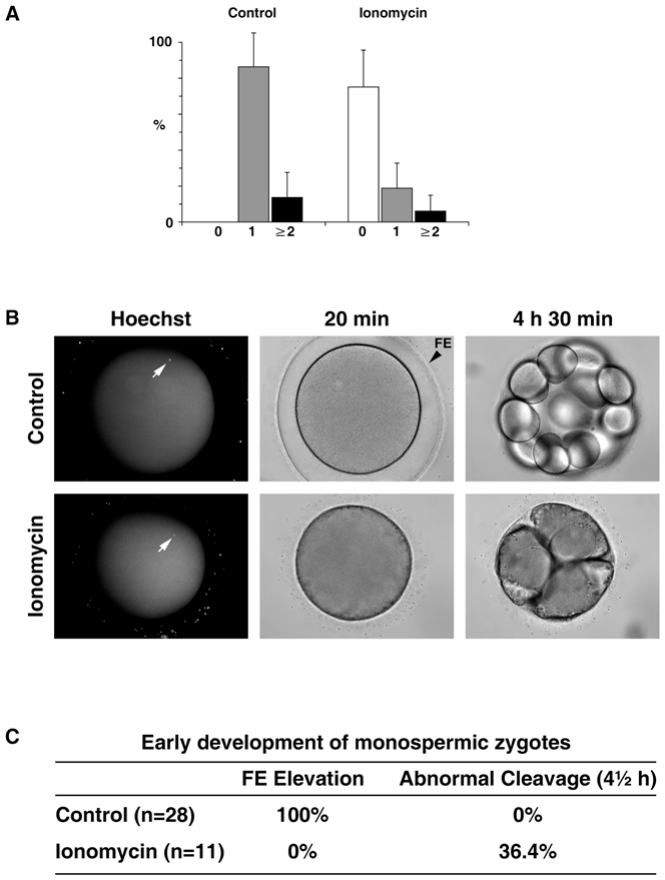
Fertilization and the early development of the ionomycin-pretreated eggs. (**A**) *Astropecten aranciacus* oocytes were pretreated with 5 µM ionomycin at the GV stage and matured with 1-MA for 1 h. Subsequently, eggs with or without (control) ionomycin pretreatment were fertilized with Hoechst 33342-stained sperm (see Materials and Methods). After 20 min, the number of the internalized sperm in each egg was counted, and the frequencies of monospermy (gray bars), polyspermy (black bars, sperm count >2), or the case with no evident sperm entry (white bar) were calculated in four independent experiments. (**B**) Developmental progress of the representative control and the ionomycin-pretreated eggs that clearly established monospermic sperm entry (arrows). (**C**) Summary of the fertilization envelope (FE) formation and the rate of abnormal development in the control and the ionomycin-pretreated eggs that established monospermic zygotes.

As shown in Fig. 2 and 4, the pretreatment of oocytes at the GV stage led to the major loss of cortical granules. Because cortical granules contribute to the formation of the fertilization envelope that is known to serve as a mechanical block to polyspermy in echinoderm eggs, the eggs matured after the brief ionomycin exposure were thought to be prone to polyspermy because of their lack of fertilization envelope. To test the idea, we have pre-stained the sperm with Hoechst 33342 and counted the number of the sperm that entered each egg following fertilization. In the given sperm concentration at fertilization, the control eggs responded mostly with monospermic fertilization (86.4±18.8%, n = 4 independent experiments), but the ionomycin-pretreated eggs were mainly with no sperm entry (75.1±20.5%, n = 4) (Fig. 9A). Thus, ionomycin-pretreated eggs appeared to have much less efficient gametes interaction that leads to sperm incorporation into the egg. For the control eggs, the frequency of monospermic fertilization far exceeds (6.4 fold) the frequency of polyspermic fertilization (13.6±18.9%, P<0.001, Turkey's test in one-way ANOVA). On the other hand, the ionomycin-pretreated eggs also displayed 3.1 fold higher rate of monospermy (18.5±14.0%) over polyspermy (6.1±8.8%), but the difference was not statistically significant. Hence, a mechanism that favors monospermic fertilization is at work in the control eggs, but the same cannot be assuredly said for the ionomycin-pretreated eggs. However, the frequency of polyspermy in the total egg population that displayed sperm entry was not significantly increased either by the ionomycin-pretreatment (20.8±19.1%, n = 3), when compared with the control (16.1±19.6%, n = 4; *p* = 0.7614). To test the effect of the ionomycin-pretreatment on early development, we followed the fate of the monospermic zygotes. Firstly, all the zygotes from the ionomycin-pretreated eggs underwent their developmental process without being shielded by the thick fertilization envelope that is normally seen in the control (Fig. 9B). Secondly, four out of eleven zygotes from the ionomycin-pretreated eggs, which had clearly displayed monospermy at fertilization, failed to develop normally at the early stages of the cell cleavage, whereas the zygotes from the monospermic control eggs all developed normally to the 16 cell stage (Fig. 9B and C). In particular, the failing zygotes from the ionomycin-pretreated eggs appeared to have a problem in creating a clear-cut cleavage furrow and thereby tended to form amorphous cell clusters (Fig. 9B). On the other hand, all polyspermic zygotes in both cases failed to develop normally (not shown).

### Ionomycin pretreatment disrupts the functionality of the cortical actin cytoskeleton

**Figure 10:**
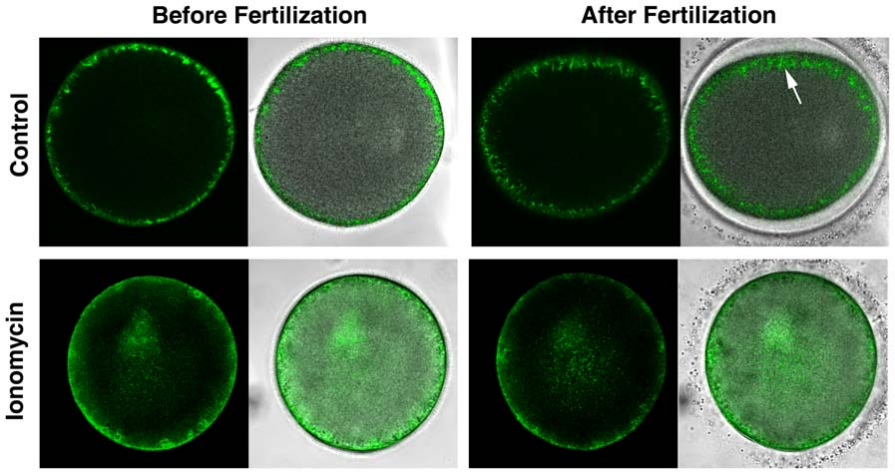
Ionomycin pretreatment disrupts the functionality of the cortical actin cytoskeleton. *P. miniata* oocytes were exposed to ionomycin (5 µM for 8 min) at the GV stage and subsequently incubated in fresh seawater containing 10 µM 1-MA. The mature eggs were then microinjected with Alexa Fluor 488-phalloidin and inseminated to monitor with confocal microscopy the real-time changes of the actin cytoskeleton. In the control eggs, the orderly arranged subplasmalemmal actin filaments migrated centripetally (arrow), which was synchronized with the elevation of the fertilization envelope. In contrast, mature eggs previously exposed to 5 µM at the GV stage failed to show such migration. At the right side of each panel, the fluorescence image of F-actin in confocal microscopy was merged with the transmission view of the same specimen. Images of the eggs before and after fertilization (13 min post-insemination) were taken from the same individual eggs.

Actin is highly implicated in the process of egg activation at fertilization. Besides Ca^2+^, the actin cytoskeleton is a decisive factor controlling exocytosis of vesicles, as has been suggested in various cell types [21], [32]–[36]. Despite the substantial increase of intracellular Ca^2+^ in response to InsP_3_ and fertilizing sperm, the ionomycin-pretreated eggs did not show any sign of vitelline layer elevation (Fig. 6 and 8), implying that the cortical changes induced by ionomycin at the GV stage of the oocytes interfered with the normal proceeding of egg activation at fertilization. In echinoderm eggs, it has been known that the subplasmalemmal actin fibers readily migrate centripetally at fertilization in concert with the elevation of the fertilization envelope [35]–[37]. In pursuit of a potential cause of the ineffective sperm entry and the tendency toward failing cleavage in the ionomycin-pretreated eggs, we have surveyed how the normal mobilization of the actin cytoskeleton is affected by the ionomycin pretreatment (Fig. 10). Whereas the control eggs at fertilization exhibited the orderly movement of the cortical actin fibers toward the center, the eggs that had been pretreated with ionomycin at the GV stage displayed neither such coordinated translocation of the ectoplasmic actin fibers nor the elevation of the fertilization envelope (Fig. 10). Hence, the brief ionomycin treatment of the oocytes at the GV stage affected not only the structure of the actin cytoskeleton but also its functionality.

### Deleterious effects of ionomycin on development

**Figure 11:**
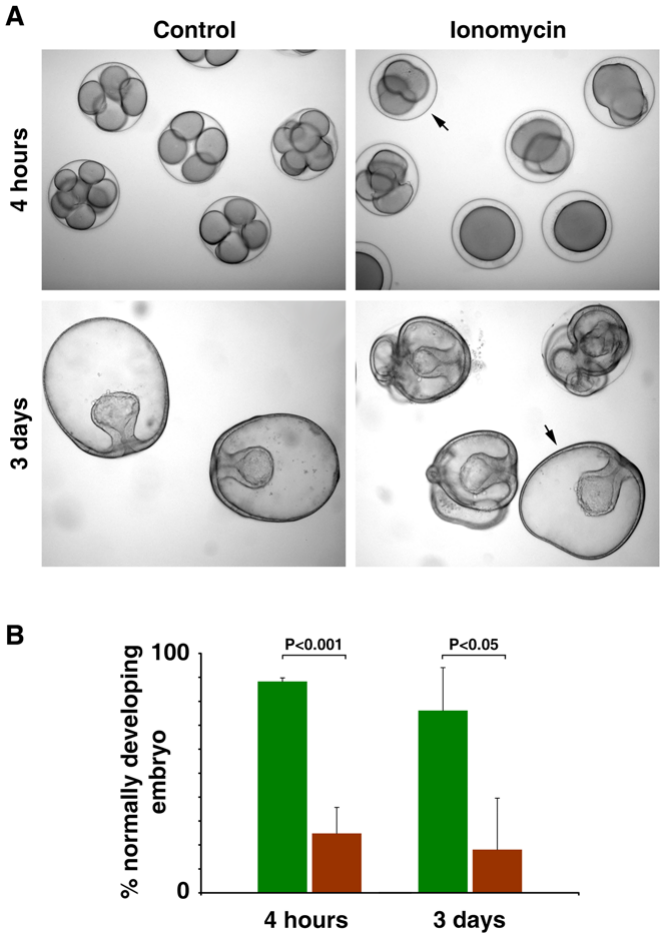
Deleterious effects of ionomycin on development. Mature eggs of *Astropecten aranciacus* were fertilized with Hoechst 33342-prestained sperm. After 20 min, zygotes displaying clear signs of monospermy and fully elevated fertilization envelope were exposed either to 5 µM ionomycin or to 0.1% DMSO (control, vehicle) for 10 min and further incubated in seawater to monitor the developmental progress. (**A**) Representative photomicrographs of the early embryos developing from the control and the monospermic zygotes exposed to ionomycin. For the latter, only the normally growing ones at 4 h and 3 d after fertilization were marked with arrows. (**B**) Frequency of normal development in the control and the ionomycin-exposed zygotes. Data were pooled from three independent batches of experiments comprising 8 to 10 monospermic zygotes with (brown bars) or without (control, green bars) the 10 min ionomycin treatment after fertilization.

To test if ionomycin affects egg activation and development of the early embryos, we have screened the fertilized eggs to select the monospermic zygotes that clearly demonstrated the entry of a single sperm and full elevation of the fertilization envelope. The monospermic zygotes were then exposed to 5 µM ionomycin for 10 min, which is close to the dose generally used on a special occasion to activate the human or animal eggs in the practice of intracytoplsmic sperm injection (ICSI) [38], [39]. The data pooled from three independent experiments indicated that, whereas 88.3±1.4% of the control zygotes developed normally 4 h after fertilization, only 24.8±10.9% of the monospermic zygotes exposed to ionomycin developed normally at 4 h (*p*<0.001) (Fig. 11). In the latter case, the majority of the zygotes was either blocked at the first cell division or displayed a problem in cell cleavage at the later stages (Fig. 11A). After 3 days, in the given experimental condition where 68.3±16.1% of the control embryos developed normally, merely 16.2±19.3% of the monospermic zygotes exposed to ionomycin after fertilization displayed normal development (*p*<0.05) (Fig. 11).

## Discussion

Since its first discovery in the 1970s, ionomycin has been widely used to increase intracellular Ca^2+^ levels in cell biology laboratories. On more rare occasions, it has also been used as a part of the standard protocol to activate the eggs that do not respond to ICSI in the *in vitro* fertilization clinics [8], [38], [39]. In this communication, by use of starfish oocytes and eggs, we have studied how egg activation and the early development are affected by the exposure to ionomycin at the GV stage or immediately after fertilization. We have found that ionomycin had detrimental effects on both egg activation and early development. First, we have demonstrated that 5 µM ionomycin readily mobilizes intracellular Ca^2+^ in the oocytes. Owing to the large cell size, we were able to visualize the spatiotemporal changes of the intracellular Ca^2+^ levels following ionomycin treatment. With the removal of the external Ca^2+^ in the media, the peak of the Ca^2+^ response by the immature oocytes were merely 53.4% of the level in the Ca^2+^-containing seawater, implying a significant contribution of the influx to the Ca^2+^ rise (Fig. 1). This discretion over the amplitude of Ca^2+^ response in the presence or absence of the external Ca^2+^ is largely alleviated in mature eggs, as the intracellular Ca^2+^ rise in CaFSW was as high as 79.0% (0.60±0.18 RFU, n = 11) of that in the ASW (0.76±0.12 RFU, n = 11) containing 10 mM Ca^2+^. Hence, with meiotic maturation, mobilization of Ca^2+^ from internal stores seems to make more contribution to the net intracellular Ca^2+^ increase by ionomycin. We have then shown that the rapid increase of intracellular Ca^2+^ was accompanied by accelerated rearrangement of the actin cytoskeleton, which exhibited depolymerization near the plasma membrane with concomitant massive polymerization and bundling in the inner cytoplasm (Fig. 3A). This is one of the most striking examples of the actin cytoskeleton being rapidly and differentially rearranged by Ca^2+^ increase in the distinct subcellular domains, but the molecular mechanisms underlying the cytoskeletal changes and their physiological significance are yet to be known. Since a certain class of actin-binding protein, e.g. gelsolin, serves as a Ca^2+^ sensor to transduce the Ca^2+^ signals into actin cytoskeletal remodeling [40], it is conceivable that similar pathway is at work to sever actin filaments or increase the barbed ends of actin filaments. Considering that actin is the most abundant (up to 300 µM) Ca^2+^-binding protein in the cytosol with unusually high binding affinity (Kd = 2–8×10^−9^ M for ATP-bound G-actin) [41], [42], and that the Ca^2+^ ion, once bound and buried in the groove of F-actin, is virtually inaccessible for exchange [43], such an extensive hyperpolymerization of actin might serve as a mechanism to alleviate the cytosolic Ca^2+^ increase at least in part [44], [45] and thereby function as a cell's adaptive defense mechanism against the potential toxicity of prolonged Ca^2+^ upheaval. On the other hand, depolymerization of actin in the subplasmalemmal region might be involved in the regulation of store-operated Ca^2+^ entry that was reported to take place in ionomycin-exposed cells [17], [46].

We have also shown that the ionomycin-induced massive Ca^2+^ increase and the drastic reorganization of the actin cytoskeleton are accompanied by the formation of the large white vesicles, which are often vested with actin filaments (Fig. 3B). This type of large (4–8 µm) vesicle was not present in the TEM of the control eggs, but appeared with the concomitant loss of smaller vesicles of the same morphology which were always present in the cortex of control oocytes. Based on the intermediate twined structure resembling two white vesicles at fusion (Fig. 2C) found in the ionomycin-pretreated oocytes but not in the control ones, we concluded that the large white vesicles were formed by fusion with other vesicles. These vesicles often contained electron-dense materials that are likely to be fragments of cortical granules, sometimes multiple in numbers (Fig. 4A, red arrow on the left). The physiological role of this type of large vesicle is unknown, although an extreme case of growing autophagic vacuoles that fuse with other vesicles or engulf cortical granules were observed in the starfish oocytes (*Pisaster ochraceus*) undergoing atresia to give room to other growing oocytes [47]. The morphology of the ‘large white vesicles’ that we observed in ionomycin-pretreated oocytes are reminiscent of the ‘clear granules’ containing residual electron-dense materials in the normal eggs of *Arbacia punctulata* [48]. These clear granules (0.83 µm in diameter) in the sea urchin eggs were identified as acidocalcisome-like organelles containing large amount of Ca^2+^ and polyphosphate. While the large white vesicles in the ionomycin-pretreated oocytes might be indicative of a cell stress after cytoskeletal rearrangement and the prolonged elevation of intracellular Ca^2+^ levels, yet to be resolved is the question about the physiological role of the ‘small’ white vesicles seen in the control oocytes.

During the brief ionomycin-induced intracellular Ca^2+^ rise, cortical granules are either exocytosed or engulfed by large white vesicles (Fig. 2 and 4). Interestingly, these eggs did not respond to a second dose of ionomycin 1 h after 1-MA addition (Fig. 4B), raising the possibility that these lost vesicles are mainly responsible for ionomycin-sensitive Ca^2+^ increase. Our result is in line with the early studies in which *Xenopus* oocytes did not produce Ca^2+^-dependent Cl^−^ currents in response to the second dose of ionomycin if the oocytes had been previously exposed to the suprathreshold (1 µM) level of ionomycin [49]. This refractory period in *Xenopus* oocytes could be overcome by 50 min of washing, but our starfish eggs did not show any sign of recovery from the refractory phase 70 min after the removal of ionomycin in the media. Thus, we were not able to establish whether the refractory phase of ionomycin is reversible in starfish eggs. In contrast to the apparent loss of the responsiveness to ionomycin, the eggs of the same treatment (5 min exposure to ionomycin at the GV stage) still responded to InsP_3_ and fertilizing sperm with substantial increase of intracellular Ca^2+^ (Fig. 6 and 7). However, the amplitude of the Ca^2+^ responses was consistently lower than in the ionomycin-untreated eggs. One possible explanation for the results is that the intracellular stores that directly contribute to the Ca^2+^ release in response to InsP_3_ or sperm were not fully recharged after the ionomycin treatment. Alternatively, we cannot rule out the possibility that the optimization of the Ca^2+^-releasing mechanisms at the intracellular stores that take place during oocyte maturation [50], [51] might have been interfered with as a result of the ionomycin pretreatment. Finally, considering that these eggs failed to respond to ionomycin and A23187 (Fig. 5), the reduction of the Ca^2+^ response might represent the fraction of the Ca^2+^ stores that are sensitive to the ionophores and InsP_3_ but had been destroyed by the ionomycin pretreatment. However, it is difficult to pinpoint which structure represents the ionomycin-disrupted InsP_3_-sensitive Ca^2+^ stores. While the conventional Ca^2+^ stores such as the endoplasmic reticulum may be accountable for them, it is also possible that the loss of the microvilli, cortical granules, and white vesicles might be linked to the reduction of the InsP_3_-dependent Ca^2+^ rise. In theory, the reduction in InsP_3_-dependent Ca^2+^ release may be ascribed to the loss of cortical granules or their associated structures. According to the early studies, cortical granules represent the major cortical Ca^2+^ stores, accounting for nearly 10% of total Ca^2+^ storage in sea urchin eggs [52]. However, the luminal Ca^2+^ of cortical granules is not likely to contribute to the intracellular Ca^2+^ increase at fertilization. Secretory vesicles of any types seem to lack InsP_3_ receptors [53], and if cortical granules possess no Ca^2+^-releasing channels, the luminal Ca^2+^ will be mostly extruded to the extracellular space at exocytosis. Nonetheless, early studies indicated that cortical granules of sea urchin eggs are tightly associated with fine network of endoplasmic reticulum that is endowed with ryanodine receptors [54]. If it also contain InsP_3_ receptors, it would be conceivable that the ionomycin-disrupted InsP_3_-sensitive Ca^2+^ store that contributes to Ca^2+^ signaling at fertilization may be the fine cisternae of endoplasmic reticulum that ensheaths the cortical granules, and not the cortical granules proper. However, the endoplasmic reticulum-enriched microsomal fraction of sea urchin egg cortices sometimes does not respond to InsP_3_ with detectable Ca^2+^ release [55]. It would be interesting to know if this fraction corresponds to the stores operated by NAADP and two-pore channels on the acidic vesicles [56]. Thus, the contribution of the Ca^2+^ pool in the cortical granules or in their associated structures still remains an open question. Alternatively, the diminished Ca^2+^ response to InsP_3_ and fertilizing sperm might reflect a reduced efficacy of the eggs' InsP_3_-dependent Ca^2+^-releasing mechanism following the extensive rearrangement of the actin cytoskeleton, as has been repeatedly suggested in the starfish oocytes and eggs [21], [35], [57], [58].

Our results in this communication raise a question about the way how the Ca^2+^-selective ionophore ionomycin works inside the cell. Intuitively, ionomycin might infiltrate the cell as a mobile ion carrier and target all the Ca^2+^ stores to transport Ca^2+^ across the membrane dissipating the concentration gradient. However, ionomycin appeared to have specific targets in exerting its impact. Firstly, the membrane fusion was apparently restricted to cortical granules and white vesicles, while yolk platelets and other organelles at the same profundity of the egg cytoplasm were not disrupted or modified by ionomycin. One possible explanation is that vesicles specializing in secretion, e.g. cortical granules and white vesicles that are normally exocytosed during fertilization (Data S1A), might be specifically vested with Ca^2+^-sensing proteins that interact with the cytoskeleton and facilitate membrane fusion, as was exemplified by synaptotagmin in synaptic vesicles [59]. Implying a role of actin cytoskeleton in the process of membrane fusion, the large white vesicles are often delineated by fluorescent probes for F-actin (Fig. 3B). Hence, it is conceivable that prolonged elevation of intracellular Ca^2+^ by ionomycin may have induced deregulated membrane fusion between these vesicles in a mechanism that involves actin. Alternatively, since the action of ionomycin is highly dependent on pH [14], such specificity might be related to the subtle difference in the luminal pH of these vesicles and organelles [60]. Secondly, the majority of InsP_3_-sensitive stores in the cortex were neither destroyed by the first exposure to 5 µM ionomycin nor responsive to the second dose of ionomycin with Ca^2+^ release. While we could not rule out that this might reflect the potential difficulties of ionomycin in diffusing inside the inner cytoplasm, we have found that ionomycin does not work when it is microinjected into the eggs (Fig. 8). Being permeant to cell membrane, ionomycin is virtually always added to the media to induce intracellular Ca^2+^ increase. Since cytosolic Ca^2+^ concentration is extremely low (10^−7^ M), our result might suggest that, to transport Ca^2+^ ions across the membrane, ionomycin should be added to the side of higher Ca^2+^ concentration. Alternatively, Ca^2+^ is transported into the cell from outside by ionomycin and this priming Ca^2+^ increase may ignite further Ca^2+^ release in an indirect mechanism either by inducing calcium-induced calcium release (CICR) or activating Ca^2+^-dependent enzymes such as phospholipase C. However, we have seen no evidence of PIP2 hydrolysis after ionomycin-induced Ca^2+^ increase inside starfish oocytes in an assay using PH-GFP (data not shown), which is at variance with the results obtained with smaller cells such as fibroblasts [61] and starfish eggs at fertilization [36]. Thus, this intriguing fundamental issue of how the ionophore ionomycin works inside the cells merits further investigation also in other experimental systems.

Finally, the results of our study indicate that the Ca^2+^ ionophore ionomycin might have a detrimental effect on both egg activation and the early embryonic development. Starfish eggs that had been briefly exposed to ionomycin at the GV stage may still undergo meiotic maturation process at least in the nucleus, but the resulting eggs displayed several problems at fertilization. First, these eggs had difficulties in producing effective sperm interaction at fertilization. While generation of the initial Ca^2+^ spot underneath the egg plasma membrane is considered as one of the indices for a meaningful sperm-egg interaction at fertilization [10], the onset of the Ca^2+^ waves in the ionomycin-pretreated eggs required more than three times longer latent period than in the control (Fig. 7A). In line with that, we have shown that the eggs pretreated with ionomycin largely lack microvilli on their surface (Fig. 2 and 4), which are known to play important roles in gametes interaction [62]. Having already lost cortical granules and other secretory vesicles (Fig. 2 and 4), the zygotes from the ionomycin-pretreated eggs failed to elevate the fertilization envelope in all cases (Fig. 9B). Furthermore, these fertilized eggs did not exhibit the characteristic centripetal migration of the cortical actin fibers that is typically seen during egg activation (Fig. 10). Hence, the ionomycin-pretreated eggs at the GV stage lacked several important hallmarks of the echinoderm egg activation. Upon sperm incorporation, the monospermic zygotes that derived from these eggs displayed further abnormality during development. Unlike the control zygotes that all normally progressed beyond the 4–16 egg stages by 4.5 h after insemination, the monospermic zygotes from ionomycin-pretreated eggs exhibited signs of failure in cell cleavage (Fig. 9B and C). Whether this abnormal development in the embryos deriving from the ionomycin-pretreated eggs is simply due to the lack of the fertilization envelope, which is thought to be protective of the developing embryo, or ascribable to the drastic alteration of the egg ectoplasm (Fig. 2 and 4) is a matter of dispute. However, it should be reminded that the monospermic zygotes that had been exposed to the same dose of ionomycin after fertilization (rather than at the GV stage) had a fully elevated fertilization envelope like the control eggs but displayed a comparable failure rate for cell cleavage and development (Fig. 11). Thus, the fertilization envelope does not seem to act as a chemical barrier to protect the embryo, and the detrimental effect of ionomycin is likely to be caused by the changes in the egg ectoplasm. There might be several different contributing factors to this, but we have focused on the actin cytoskeleton. Being dynamically regulated upon cell signals, the actin cytoskeleton is indeed implicated in many aspects of fertilization and early development [45]. As demonstrated in sea urchin, the cell cleavage of zygotes heavily depends on the regulation of the actin cytoskeleton [63]. The actin filaments in the egg cortex even extend through the perivitelline space of the fertilization envelope [36]. Hence, actin is intimately implicated in fertilization and in the early stages of development. We have shown that the elevated rate of aberrant development in the ionomycin-treated eggs is preceded by the drastic alteration of the actin cytoskeleton in the egg cortex and the retraction of microvilli (Fig. 2–4), as well as the failure of the centripetal migration of the cortical actin filaments (Fig. 10). Finally, it bears an emphasis that it is difficult to extrapolate our findings to make a direct comparison with the similar use of ionomycin in the rare case of the medical practice of ICSI in the *in vitro* fertilization clinics [64]. The assessment on the safety of such procedure awaits more detailed studies using the zygotes being produced with the same procedure, e.g. intracytoplasmic sperm injection.

## Materials and Methods

### Ethics statements

No specific permits were required for the described field studies for starfish. Field studies did not involve endangered or protected species nor private territory for sample collection.

### Preparation of oocytes and reagents

Captured starfish (*Astropecten aranciacus* and *Pisaster miniata*) were maintained in circulating cold seawater (16°C). The female gonads were dissected from the central dorsal area near the arms and transferred to the filter-sterilized cold seawater. Free individual oocytes were isolated by repeatedly passing through gauze and rinsing in cold filtered seawater. Oocytes were collected by low speed (<1,000 rpm) sedimentation in each step. Immature oocytes obtained in this way were marked by the presence of the large nucleus (germinal vesicles). Ionomycin and InsP_3_ were purchased from Invitrogen. All other chemicals were purchased from Sigma-Aldrich unless stated otherwise, and all solutions were prepared following the manufacturers' instruction. Certain experiments were performed in artificial seawater (ASW: 490 mM NaCl, 8 mM KCl, 10 mM CaCl_2_, 12 mM MgCl_2_, 2.5 mM NaHCO_3_, pH 8.0) or Ca^2+^-free seawater (500 mM NaCl, 8 mM KCl, 12 mM MgCl_2_, 2.5 mM NaHCO_3_, 2 mM EGTA, pH 8.0). Hoechst 33342 was purchased from Sigma and the final concentration of 5 µM was used to stain sperm for 1 min before insemination. The number of egg-incorporated sperm was counted in epifluorescence microscopy.

### Microinjection, caged compounds, and video imaging

Microinjection of the oocytes was performed with an air-pressure Transjector (Eppendorf), following the procedure that was previously described in detail [36]. Fluorescent calcium dye (Calcium Green) conjugated with 10 kDa dextran (Molecular Probes) was used in 500 µM (pipette concentration). The injection buffer contained 10 mM Hepes and 100 mM L-Asp at pH 7.0. To activate the caged InsP_3_ (Molecular Probes), microinjected eggs were irradiated with 330 nm UV light by using the computer-controlled shutter system Lambda 10-2 (Sutter Instruments, Co., Novato, CA). Alexa Fluor 488-phalloidin (Molecular Probes) was injected as described previously [35]. All Ca^2+^ imaging work in this study was done by use of epifluorescence microscopy. Cytosolic Ca^2+^ changes were detected using a cooled CCD camera (MicroMax, Princeton Instruments, Inc., Trenton, NJ) mounted on a Zeiss Axiovert 200 microscope with a Plan-Neofluar 20x/0.50 objective. The quantified Ca^2+^ signal at a given time point was normalized to the baseline fluorescence (F_0_) following the formula F_rel_ = [F−F_0_]/F_0_, where F represents the average fluorescence level of the entire oocyte. Fluorescence of Ca^2+^ images were analyzed with the MetaMorph Imaging System software (Universal Imaging Corporation, West Chester, PA).

### Statistical analysis

The numerical MetaMorph data were compiled and analyzed with Excel of Microsoft Office 2003. The average and variation of the data were reported as ‘mean ± standard deviation (SD)’ in all cases. The paired t-test and the one-way ANOVA were performed by use of Prism 3.0 (GraphPad Software, La Jolla, CA, USA), and the P-values smaller than 0.05 (P<0.05) were considered statistically significant.

### Light and Transmission electron microscopy (TEM)

Starfish oocytes and eggs to be analyzed following the ionomycin and 1-MA treatment were fixed together with proper control samples in filtered seawater containing 1% glutaraldehyde (pH 8.0) for 1 h at room temperature and then post-fixed with 1% osmium tetroxide for 1 h. Specimens were dehydrated in increasing concentrations of alcohol and embedded in EPON 812. The polymerized resin containing the fixed material was sectioned in two ways. Semi-thin (1 micron) sections were stained with toluidine blue and examined by light microscopy with a Zeiss Axiovert 200 microscope, and the images were captured by the CCD camera. For TEM, ultrathin sections were stained with 2% uranyl acetate and 0.2% lead citrate, and examined with a LEO 912 AB energy filter transmission electron microscope.

### Confocal Microscopy

GV stage oocytes and eggs microinjected with Alexa Fluor 488–conjugated phalloidin were viewed with a Zeiss LSM 510 META Laser Scanning Confocal Microscope (Jena, Germany) with excitation at 488 nm and emission at 500/555 nm. A Plan-Neofluar 25x/0.80 objective water lens was used to produce optical slices from the specimens. The images were captured as digital computer files and examination of the fluorescence was performed using MetaMorph image analysis software.

## Supporting Information

Data S1 **Translocation of white vesicles during egg maturation and activation.** (**A**) Light microscope images of immature oocytes of *A. aranciacus* show that white vesicles are mainly located in the cortex but not tightly packed underneath the plasma membrane (white arrows). In sharp contrast, white vesicles in the mature eggs are closely associated with plasma membrane just like cortical granules. Note that the white vesicles are largely eliminated in the activated egg that underwent massive exocytosis and the elevation of the vitelline layer. (**B**) TEM image of white vesicles containing remnants of fibrillary contents in the normal eggs of *Astropecten aranciacus* at fertilization. As a result of cortical granules (black arrow) exocytosis and microvilli (gray arrow) elongation, the fertilization envelope was fully elevated (labeled as VL, vitelline layer). PS, perivitelline space. The white vesicle fusing with the plasma membrane is marked by a white arrow. Scale bar 1 µm.
(PDF)
