# Supplementary figures and images for: Effects of Ionomycin on Egg Activation and Early Development in Starfish
Source: PLoS One. 2012 Jun 18;7(6):e39231. doi: 10.1371/journal.pone.0039231 (PMC3377674; doi:10.1371/journal.pone.0039231)

**A****Immature  
Oocyte**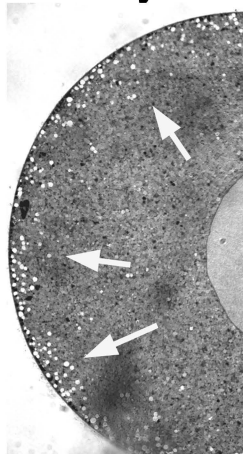**Mature Egg**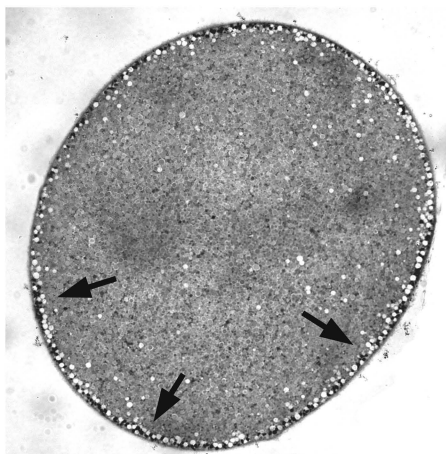**Activated Egg**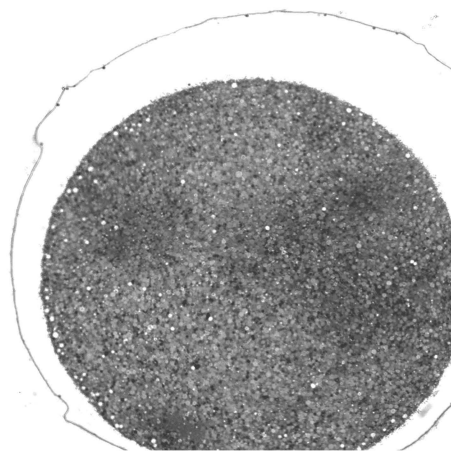**B**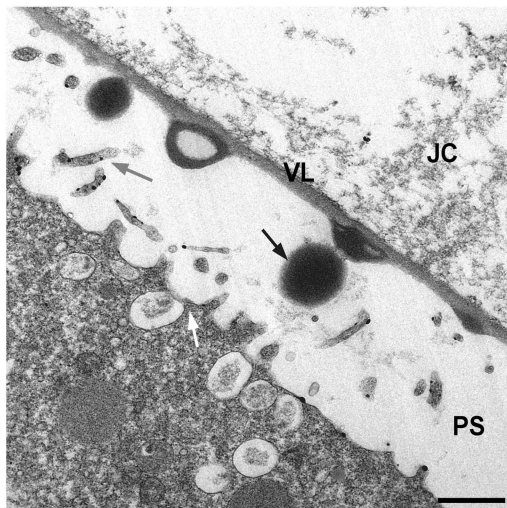

Supplement: Data S1 — Translocation of white vesicles during egg maturation and activation. (A) Light microscope images of immature oocytes of A. aranciacus show that white vesicles are mainly located in the cortex but not tightly packed underneath the plasma membrane (white arrows). In sharp contrast, white vesicles in the mature eggs are closely associated with plasma membrane just like cortical granules. Note that the white vesicles are largely eliminated in the activated egg that underwent massive exocytosis and the elevation of the vitelline layer. (B) TEM image of white vesicles containing remnants of fibrillary contents in the normal eggs of Astropecten aranciacus at fertilization. As a result of cortical granules (black arrow) exocytosis and microvilli (gray arrow) elongation, the fertilization envelope was fully elevated (labeled as VL, vitelline layer). PS, perivitelline space. The white vesicle fusing with the plasma membrane is marked by a white arrow. Scale bar 1 µm. (PDF) [file pone.0039231.s001.pdf]
